# Rescue bedside laparotomy in the intensive care unit in patients too unstable for transport to the operating room

**DOI:** 10.1186/cc13925

**Published:** 2014-06-16

**Authors:** Joerg Schreiber, Axel Nierhaus, Eik Vettorazzi, Stephan A Braune, Daniel P Frings, Yogesh Vashist, Jakob R Izbicki, Stefan Kluge

**Affiliations:** 1 Department of Intensive Care Medicine, University Medical Center Hamburg-Eppendorf, Martinistr. 52, 20246 Hamburg, Germany; 2 Institute for Biometry and Epidemiology, University Medical Center Hamburg-Eppendorf, Martinistr. 52, 20246 Hamburg, Germany; 3 Department of General, Visceral and Thoracic Surgery, University Medical Center Hamburg-Eppendorf, Martinistr. 52, 20246 Hamburg, Germany

## Abstract

**Introduction:**

The prognoses of critically ill patients with a requirement for emergency laparotomy and severe respiratory and/or hemodynamic instability precluding transport to the operating room (OR) are often fatal without surgery. Attempting emergency surgery at the bedside might equally result in an adverse outcome. However, risk factors and predictors that could support clinical decision making have not been identified so far. This study describes the clinical characteristics, indicative pathophysiology and outcomes in patients undergoing resuscitative laparotomy in the intensive care unit (ICU).

**Methods:**

This was a retrospective observational study of all critically ill adult patients undergoing resuscitative laparotomy in the ICUs of a German university hospital from January 2005 to July 2013. Clinical characteristics, risk factors, and treatments were compared between survivors and non-survivors. The primary endpoint was 28-day survival.

**Results:**

A total of 41 patients with a median age of 64 (21 to 83) were included. The most frequent reasons for ICU admission were sepsis, pneumonia, and pancreatic surgery. All patients were mechanically ventilated, receiving vasopressors, and were in multiple organ failure. Twenty-nine patients (70.7%) were on renal replacement therapy and two patients (4.9%) on extracorporeal membrane oxygenation. The main reasons for surgery were suspected intra-abdominal bleeding (39.0%), suspected intestinal ischemia (24.4%) or abdominal compartment syndrome (24.4%). Twenty-eight-day, ICU and hospital mortalities were 75.6%, 80.5%, and 82.9%, respectively. In six out of ten patients (60%) who survived surgery for more than 28 days, bedside laparotomy was rated as a life-saving procedure by an interdisciplinary group of the investigators.

**Conclusions:**

These findings suggest that in selected critically ill patients with a vital indication for emergency laparotomy and severe cardiopulmonary instability precluding transport to the OR, a bedside resuscitative laparotomy in the ICU can be considered as a rescue procedure, even though very high mortality is to be expected.

## Introduction

In general, elective surgery is performed in specially equipped operating rooms (ORs). Optimal hygiene, lowering the rate of perioperative infections, the presence of specialized personnel, optimized lighting conditions and the availability of all essential surgical equipment and infrastructure make the OR the optimal site for performing surgery [1].

It has been shown that some elective surgical procedures, such as tracheostomies [2,3], percutaneous endoscopic gastrostomy [4] and open lung biopsy [5] can be safely performed at the bedside in the intensive care unit (ICU). In such cases, avoiding transfer to the OR offers an advantage without any negative consequences. In patients with acute and life-threatening intra-abdominal conditions and vital indication for emergency laparotomy, but with severe hemodynamic and/or respiratory instability precluding safe transport to the OR, bedside rescue laparotomy in the ICU might be the only viable intervention [6]. Clinical scenarios necessitating emergency surgery include major uncontrollable intra-abdominal hemorrhage, acute intestinal ischemia or abdominal compartment syndrome (ACS) leading to and/or being caused by severe cardiopulmonary instability [7,8].

At present there is only limited data on bedside emergency surgery outside the OR. Few studies have analyzed bedside laparoscopy in critically ill patients with acute abdomen [9,10] or emergency thoracotomy in the emergency room [11,12]. Reports on resuscitative laparotomy in the ICU are scarce and have analyzed heterogeneous cohorts with respect to the degree of urgency (emergency and elective surgery) [13,14] or with respect to the instability and the site of surgery (surgery in the ICU and in the OR) [15-18]. Attempting emergency surgery at the bedside might equally result in an adverse outcome. However, risk factors and predictors that could support clinical decision making have not been identified so far. Therefore, the aim of this study was to describe the clinical characteristics, pathophysiological indicators, and outcomes of a cohort of severely ill adult patients all too unstable for transport to the OR undergoing emergency bedside laparotomy as a vital indication in the ICU. We hypothesized that (1) in these patients, even though a very high mortality rate will be unavoidable, attempting emergency surgery is not futile, and (2) that we would be able to identify pathophysiological markers indicating poor outcome.

## Material and methods

### Study design

This retrospective observational study included all adult critically ill patients treated in the Department of Intensive Care Medicine of the University Medical Center Hamburg-Eppendorf between January 2005 and July 2013 who underwent bedside resuscitative emergency laparotomy. The Department of Intensive Care Medicine at the University Medical Center Hamburg-Eppendorf, Germany, consists of 11 ICUs with a total of 132 beds and provides intensive care medicine to all adult patients in the university hospital. Approximately 7,000 patients are treated annually in the department (data from 2012). All patients were situated either in a single or two-bed room at the time of bedside surgery. Patients were included only if their highly compromised hemodynamic and/or respiratory state precluded a transfer from the ICU to the OR. This decision was reached by an interdisciplinary consensus by senior staff members in all cases if (1) the patient required more than 1.2 μg/kg/min of norepinephrine, or (2) was on 100% oxygen with a positive end-expiratory pressure (PEEP) level of more than 14 mbar resulting in a fraction of inspired oxygen/arterial oxygen tension (F_i_O_2_/P_a_O_2_) ratio below 150 mmHg and a documented critical deterioration of oxygenation if derecruitment occurred, or (3) was on extracorporeal life support (extracorporeal membrane oxygenation (ECMO), intra-aortic balloon pump (IABP)). Patients who underwent elective bedside abdominal surgery as an exception due to internal logistic reasons like staff shortage in the OR were excluded, as were all ICU cases with bedside laparoscopy. Primary endpoint was 28-day survival.

Assessing the vital indication for emergency laparotomy and the risks of transport to the OR in these patients and performing a bedside resuscitative laparotomy in the ICU was a highly individualized and collective decisional process of the senior consultants of the Department of Intensive Care Medicine and the Department of Surgery.

The ethics committee of the Hamburg Chamber of Physicians approved the collection, analysis, and publication of the retrospectively obtained and anonymized data and waived the need to obtain informed consent for this non-interventional study.

### Data collection

In January 2005, the Department of Intensive Care Medicine was founded at our University Medical Center, consisting of all adult ICU beds in the hospital. At the same time, a comprehensive patient data management system was installed, giving access to the complete medical record of the patients. For reasons of data quality and integrity we defined this point in time as the effective date to start the observation period.

From the electronic patient data management system (Integrated Care Manager ICM, Draeger Medical, Lübeck, Germany) the following data were obtained: demographics, clinical characteristics, laboratory findings, medications, simplified acute physiology score II (SAPS II), acute physiology and chronic health evaluation II (APACHE II) score, indications for emergency laparotomy, intraoperative findings, type and duration of surgery, surgery-related complications, and length of stay (LOS) in the ICU and in the hospital. To assess the effect of bedside surgery in the 28-day survivors (n = 10), all authors individually categorized surgery in the survivor cases as ‘life-saving surgery’, ‘with unclear surgical impact on immediate survival’, or ‘without life-saving surgical impact’. The cases were analyzed taking into account diagnosis, intraoperative findings and the postoperative results. We discussed this in depth among the authors. In the case of disagreement (two cases) a consensus was reached through group discussion.

### Statistical analysis

Continuous variables are expressed as medians (with range). Categorical variables are expressed as counts (and percentages). In order to compare the clinical characteristics and risk factors between survivors and non-survivors, non-parametric analyses were performed. Depending on the data type *χ*^2^ test, Fisher’s exact test, or Mann-Whitney *U* test were applied. A two-sided *P* value of <0.05 was considered significant. Receiver operating characteristic area under the curve according to de Long was performed. The software used for analyses was IBM SPSS 20.0 (IBM Corp., Armonk, NY, USA).

## Results

### Patient characteristics

During the study period, 41 critically ill patients with a median age of 64 years underwent resuscitative bedside laparotomy in the ICU. Table 1 shows the cohort’s clinical characteristics on ICU admission. The most frequent diagnoses leading to ICU admission were sepsis (24.4%), pneumonia (14.6%), and postoperative admission after pancreatic surgery (12.2%).

**Table 1:** Reasons for ICU admission

**Diagnosis on admission**	**n (%)**
Sepsis	10 (24)
Pneumonia	6 (15)
Pancreatic surgery	5 (12)
Liver transplantation	4 (10)
Intra-abdominal bleeding	4 (10)
CPR	3 (7)
ARDS	2 (5)
Trauma	2 (5)
Other	5 (12)

CPR, cardiopulmonary resuscitation; ARDS, acute respiratory distress syndrome.

At the time of resuscitative laparotomy all patients were mechanically ventilated with a median PaO_2_/FiO_2_ ratio of 136 mmHg (33 to 559). The median dose of norepinephrine was 1.17 (0.02 to 11.33) μg/kg/min. Thirty-seven patients (90.2%) had acute renal failure and 29 (70.7%) received renal replacement therapy; two patients (4.9%) were on ECMO and one patient was treated with an IABP. Of the 41 patients, 20 (48.7%) were ventilated with a F_i_O_2_ of 1.0 and a PEEP level of above 14 mbar. Eighteen patients (44%) received norepinephrine at a dose of above 1.2 μg/kg/min, three patients (7.3%) had extracorporeal devices. Further clinical characteristics on the day of surgery are given in Table 2.

**Table 2:** Demographic and clinical data of survivors (at 28 days) and non-survivors

		**Survivors**	**Non-survivors**	***P *** **value**
		**n = 10 (24.4%)**	**n = 31 (75.6%)**	
Age		53.5 (21-78)	64 (27-83)	0.387
Sex (female/male)		6/60	4/12.9	0.006
SAPS II (ICU admission)		43 (32-74)	60 (30-85)	0.027
SAPS II (day of surgery)		44 (33-72)	67 (41-83)	0.001
APACHE II score (day of admission)		17 (12-38)	29 (9-42)	0.039
APACHE II score (day of surgery)		18 (12-34)	31 (12-44)	0.002
Time from ICU admission to surgery	days	5 (0-56)	2 (0-26)	0.342
Length of stay in ICU post-op	days	48 (14-68)		
***Laboratory data***				
Hemoglobin	g/dL	9.7 (6.3-13,4)	8.0 (2.4-15.1)	0.012
WBC	x10^3^/μL	13.1 (1.0-85.5)	13.6 (0.3-32.2)	0.832
Platelets	x10^3^/μL	119 (34-405)	62 (12-620)	0.202
International normalized ratio		1.29 (1.03-2.46)	1.49 (0.96-3.43)	0.208
Alanine aminotransferase	IU/L	45 (6-1350)	430 (6-6936)	0.024
Serum creatinine	mg/L	1.31 (0.4-2.4)	1.7 (0.8-7.7)	0.060
C-reactive protein	mg/L	56 (10-337)	115 (<5-504)	0.820
Procalcitonin	μg/L	3.63 (0.2-263)	7.4 (0.93-292)	0.896
Lactate	mmol/L	3.3 (2.4-10.7)	11.4 (2.9-27.0)	<0.001
pH		7.31 (7.21-7.47)	7.14 (6.87-7.51)	<0.001
Base excess	mEq/l	−1.0 (−8.1-3.1)	−13.1 (−21.3-4.4)	<0.001
S_cv_O_2_	%	74 (63-84)	71 (38-86)	0.436
***Gas exchange***				
Mechanical ventilation				
F_i_O_2_		0.85 (0.3-1.0)	0.9 (0.3-1.0)	0.923
P_a_O_2_/F_i_O_2_ ratio	mmHg	171 (33-503)	129 (49-559)	0.835
P_a_CO_2_	mmHg	51 (33-67)	48 (29-90)	0.821
***Renal situation***				
Acute renal failure		10 (100%)	27 (87.1%)	1.0
Renal replacement therapy		8 (80%)	21 (77.4%)	0.725
***Circulation***				
*Vasopressor*				
Norepinephrine mcg/kg/min		0.6 (0.02-1.88)	1.37 (0.12-11.3)	0.039
IABP				1.0
***Surgery***		0 (0%)	1 (3.2%)	
Duration of surgery	minutes			0.553
***Transfusion***		45 (19-190)	40 (10-92)	
Number of red cell transfusions 24 h pre-op		1 (0-6)	2 (0-105)	0.253
Number of red cell transfusions intra/24 h post-op		3 (0-12)	4 (0-50)	0.535

Values given as median (range) or number (%); *P* values from Mann-Whitney *U* test for continuous data, Fisher’s exact test is used for categorical data. SAPS II, simplified acute physiology score II; APACHE II, acute physiology and chronic health evaluation II; WBC, white blood cell count; S_cv_O_2_, central venous oxygen saturation; F_i_O_2_, fraction of inspired oxygen; P_a_O_2_, arterial oxygen tension; P_a_CO_2_, arterial carbon dioxide tension; ECMO, extracorporeal membrane oxygenation; IABP, intra-aortic balloon pump; pre-op, preoperative; post-op, postoperative.

### Surgical procedures

The reasons for laparotomy were: suspected abdominal bleeding (n = 16; 39%), suspected intestinal ischemia (n = 10; 24.4%), abdominal compartment syndrome (n = 10; 24.4%) or a suspected intra-abdominal focus for sepsis (n = 5; 12.2%). The 10 subjects with abdominal compartment syndrome had an increased median abdominal pressure of 27 cmH_2_O (range 20 to 180) as measured indirectly by determining intravesical pressure. The median duration of surgery was 40 minutes (10 to 190). In 33 (80.5%) of the 41 cases the suspected diagnosis was confirmed intraoperatively, only five cases (12.2%) yielded a negative result. Fourteen patients (34.1%) required a subsequent surgical intervention in the OR and all of these patients had a temporary abdominal closure. Temporary closure was achieved by either the insertion of a Vicryl woven mesh or by vacuum-assisted closure (VAC). Seven patients (17.1%) underwent a postmortem examination. All autopsy findings were in line with the suspected diagnoses and the surgical findings. No obvious major surgical complications, such as iatrogenic injury, uncontrollable bleeding, sepsis caused by wound infection, or death occurred in any of the 41 patients. Table 3 provides further details of the surgical procedures.

**Table 3:** Suspected and intraoperative diagnoses, surgical procedures

**Indication for surgery/suspected diagnosis**	**n (%)**
Intra-abdominal bleeding	16 (39.0)
Intestinal ischemia	10 (24.4)
Abdominal compartment syndrome	10 (24.4)
Abdominal focus of sepsis	5 (12.2)
**Intraoperative diagnosis**	
Intra-abdominal bleeding	17 (41.5)
diffuse bleeding	7
venous bleeding (mesenteric vein; portal vein; gastric vein, vena cava)	4
hepatic bleeding	2
arterial bleeding (gastroduodenal artery; aorta)	2
vascular anastomotic repair bleeding	2
Intestinal ischemia	9 (22.0)
Abdominal compartment syndrome	8 (19.5)
Anastomotic breakdown	2 (4.9)
Negative finding	5 (12.2)
Patients needing subsequent surgery in OR	14 (34.1)
**Surgical procedures**	
Surgical control of bleeding	11 (26.8)
Decompressive laparotomy	10 (24.4)
Packing	8 (19.5)
Vascular repair	3 (7.3)
Intestinal resection	9 (21.9)
Peritoneal lavage, focus control	5 (7.3)

OR, operating room.

### Outcomes

The 28-day, ICU, and hospital mortality was 75.6%, 80.5%, and 82.9%, respectively. The median time from ICU admission to resuscitative laparotomy was three days (0/56).

Table 2 shows the clinical characteristics of the 28-day survivors and non-survivors. No patient with a suspected diagnosis of intestinal ischemia (n = 10) survived. Non-survivors had significantly higher SAPS II and APACHE II scores, required significantly higher doses of norepinephrine, and had significantly lower pH and higher serum lactate values. No patient with lactate >10.7 mmol/l or pH less than 7.21 survived. When calculating receiver operating characteristic area under the curve and the odds ratio for non-survival, it became evident that the variables indicating severe metabolic derangement and acidosis (base excess, lactate and pH) had the highest predictive power (Table 4). Twenty-eight-day survivors (24.4%) had higher median hemoglobin concentrations, but did not differ significantly from the non-survivors regarding the number of transfused red blood cell units 24 hours before and after surgery. There was no statistically significant difference between survivors and non-survivors regarding laboratory markers of inflammation or sepsis (white blood cell count, C-reactive protein and procalcitonin). Neither P_a_O_2_/F_i_O_2_ ratios nor arterial carbon dioxide tension (P_a_CO_2_) levels differed significantly between the groups.

**Table 4:** Receiver operating characteristic and odds ratio for non-survival

**Parameter**	**ROC-AUC (95% CI)**	**Odds ratio (95% CI)**
Base excess	0.92 (0.83, 1.00)	70 (6.3, 777.87)^1^
Lactate	0.92 (0.82, 1.00)	56.25 (5.53, 572.39)^2^
pH	0.85 (0.74, 0.97)	15.75 (2.84, 87.24)^3^
SAPS II (day of surgery)	0.84 (0.68, 1.00)	8 (1.62, 39.3)^4^
APACHE II (day of surgery)	0.82 (0.67, 0.98)	1.18 (1.05, 1.32)^5^
Norepinephrine (mcg/kg/min)	0.78 (0.62, 0.94)	3 (0.69, 13.1)^6^

Base excess <2 mEq/l; lactate >5 mmol/l; pH <7.3; SAPS II >55; APACHE II >25; norepinephrine dose >1 μg/kg/min. ROC-AUC, receiver operating characteristic area under the curve with confidence interval (CI) according to de Long; SAPS II, simplified acute physiology score II; APACHE II, acute physiology and chronic health evaluation II.

Of the 10 patients who survived 28 days, the emergency surgery was considered a life-saving procedure in six cases (60%) by the investigators. Two of these six patients required cardiopulmonary resuscitation within 24 hours before surgery.

## Discussion

We report the clinical characteristics and outcomes of a cohort of 41 critically ill patients with severe multiorgan failure who underwent resuscitative bedside laparotomy for vital indications. Emergency surgery was performed in the ICU because these patients were considered too unstable for transport to the OR and their short-term prognosis was deemed lethal without emergency surgery. No relevant surgically related complications were observed.

For a variety of reasons, the ICU is increasingly being used as a place for surgical procedures that were traditionally done in the OR [19]. Examples of (semi-)elective bedside surgery are the percutaneous dilatational tracheostomy [20] and percutaneous endoscopic gastrostomy [21]. Many surgical centers increasingly perform routine laparotomies in critically ill patients [22].

Laparoscopy is another surgical procedure that has been reported to be performed at the bedside in ICU in patients with acute abdomen [10,23,24]. A recent study on 62 critically ill patients showed bedside laparoscopy to be useful for obtaining a diagnosis in patients with acute abdomen [9]. However, in cases with positive findings (69%), all patients had sufficient cardiopulmonary stability for transfer to the OR where they eventually underwent open surgery. Bedside laparoscopy, although less invasive than open surgery, not only has therapeutic but also diagnostic limitations. Early stages of mesenteric ischemia are difficult to verify and the sensitivity for retroperitoneal pathology is low [9,10]. Moreover, in the case of highly urgent surgery, for example for acute bleeding, setting up a laparoscopy is often too time-consuming and therefore not an adequate approach. Another limitation is capnoperitoneum potentially further compromising the hemodynamics of already unstable patients.

As opposed to elective surgery, bedside resuscitative laparotomy in the ICU is considered as a ‘heroic procedure’ for patients who are *in extremis* and not transferable to the OR. Thus data for emergency laparotomies outside the OR are scarce and mostly refer to decompressive laparotomy in refractory ACS, for example in burns patients [15] or in trauma patients [13,14,25]. Unlike emergency bedside laparotomy, there are good data for emergency laparotomy in the OR, especially regarding the outcome of patients [8]. In a large cohort of critically ill patients who underwent emergency laparotomy, the mortality was 30.7%, but these patients were stable enough to be operated in the OR with only postoperative ICU admission [26]. Using a protocol for bedside laparotomy Diaz *et al*. described 60 [14] and 75 [13] patients who underwent bedside laparotomy in a trauma ICU. Mortality rates were 23.3% and 49%, respectively and the authors concluded that bedside laparotomy is feasible, safe, and carries an acceptable risk of complications and death considering the severity of illness of these patients. However, these studies mainly included trauma patients who underwent both elective surgical procedures (peritoneal lavage, closure of abdomen) as well as emergency procedures.

Lund *et al*. explored the outcome of trauma patients with hypovolemic shock who underwent emergency laparotomy in the emergency room due to hemodynamic instability [27]. The 30-day mortality rate of 44 patients with penetrating or blunt abdominal trauma was 59%. Struck *et al*. recently reported on a cohort of 35 burns patients with ACS undergoing emergency laparotomy [15]. Overall mortality was 71.4%, but only six patients underwent a bedside laparotomy in the ICU. Despite similar overall mortality rates, all 41 patients in our cohort were considered too unstable for transport.

Apart from the Struck *et al*. study, the 28-day mortality rate of 75.6% in our cohort of 41 patients undergoing resuscitative bedside laparotomy was higher than in the other above mentioned studies. The most likely reason for this is the greater severity of illness in our cohort. All previous studies on laparotomy in the ICU were in trauma patients, who are highly underrepresented in our cohort. By the day of ICU admission the median SAPS II of our cohort was already as high as 58 with a predicted mortality of 64.0% [28], similar to the mortality of 60.5% predicted from the median APACHE II score of 27 [29]. In addition, during the course in ICU their clinical state further deteriorated due to progressive multiorgan failure and/or secondary complications, subsequently increasing their mortality risk. This explanation is underlined by the fact that within 24 hours of requiring emergency surgery the SAPS II and APACHE II score increased and as many as 13 patients (31.7%) required cardiopulmonary resuscitation.

One aim of our study was to identify predictive factors for survival after resuscitative bedside laparotomy. As one would expect, severe metabolic derangements were most predictive for adverse outcome. We found that no patient with intestinal ischemia, with lactate >10.7 mmol/l or pH <7.21 survived. Accordingly, the markers of metabolic acidosis showed the highest odds ratios for non-survival (Table 4). However, due to our small sample size we do not suggest that diagnosis of intestinal ischemia or certain laboratory values should be used to exclude patients for resuscitative bedside surgery.

At the time of surgery, all of our critically ill patients were unable to communicate, making a meaningful physical examination of the abdomen or thorax difficult. Unlike readily performed bedside procedures, such as laboratory tests, ultrasound or plain X-rays, more complex imaging techniques, such as computer and/or magnetic resonance tomography necessitate transfer out of the ICU. By definition, all of our patients were considered to have insufficient cardiopulmonary stability for any transfer and had a presumed fatal prognosis without surgery. The decision to perform immediate and resuscitative laparotomy in the ICU was not based on any standard operating procedures but on a highly individualized joint decision of the senior consultants of the Department of Intensive Care and the Department of Surgery.

Our study has some methodological limitations, which mainly arise from its design. First, the interpretation of the results is limited by potential biases introduced by the retrospective analysis and the inherent methodological lack of a control group in this type of clinical scenario. Ethical considerations and critical timing in emergency situations probably preclude the performance of randomized trials in this very challenging setting. However, we postulate that severe and life-threatening complications, because of their clinical impact, were most likely fully documented at the time of occurrence and detected during data acquisition. Second, the rare but severe clinical scenario limits the sample size despite the long study period. Finally, we cannot assess how many patients in a similar clinical scenario, who were not operated on and who subsequently died, could potentially have been saved by an emergency bedside laparotomy.

## Conclusions

The results of our study suggest that in extreme situations with a vital indication for emergency laparotomy but severe cardiopulmonary instability preventing transport to the OR a rescue laparotomy in the ICU should be considered as a potential treatment option as a last resort. This applies especially to cases where death is imminent without immediate surgery.

## Key messages

• Some critically ill patients are highly cardiopulmonary instable, precluding transport to the OR.

• In selected critically ill patients with a vital indication for emergency laparotomy and severe cardiopulmonary instability, precluding transport to the OR, a bedside rescue laparotomy in the ICU can be considered even though it is associated with a very high mortality, especially in patients with a profound metabolic acidosis.

## Abbreviations

ACS: abdominal compartment syndrome; APACHE: acute physiology and chronic health evaluation; ECMO: extracorporeal membrane oxygenation; F_i_O_2_: fraction of inspired oxygen; IABP: intra-aortic balloon pump; ICU: intensive care unit; LOS: length of stay; OR: operating room; P_a_O_2_: arterial oxygen tension; P_a_CO_2_: arterial carbon dioxide tension; PEEP: positive end-expiratory pressure; SAPS: simplified acute physiology score; VAC: vacuum-assisted closure.

## Competing interests

The authors declare that they have no competing interests.

## Authors’ contributions

JS, AN, SB and SK have made substantial contributions to conception and design of the study as well as to the acquisition, analysis and interpretation of data. AN, DF, YV and JI have revised it critically for important intellectual content and have made substantial contributions to analysis and interpretation of data. EV has revised the statistical results and added important statistical content. JS, AN, SB and SK have drafted the submitted manuscript. AN has edited, revised and partially rewritten the manuscript. All authors read and approved the final manuscript.
